# Context conditioning and extinction in humans: differential contribution of the hippocampus, amygdala and prefrontal cortex

**DOI:** 10.1111/j.1460-9568.2009.06624.x

**Published:** 2009-02

**Authors:** Simone Lang, Alexander Kroll, Slawomira J Lipinski, Michèle Wessa, Stephanie Ridder, Christoph Christmann, Lothar R Schad, Herta Flor

**Affiliations:** 1 Department of Clinical and Cognitive Neuroscience, Central Institute of Mental Health, University of HeidelbergMannheim, Germany; 2 Department of Computer Assisted Clinical Medicine, Faculty of Medicine Mannheim, University of HeidelbergMannheim, Germany

**Keywords:** anxiety, fear learning, functional connectivity, functional magnetic resonance imaging (fMRI)

## Abstract

Functional magnetic resonance imaging was used to investigate the role of the hippocampus, amygdala and medial prefrontal cortex (mPFC) in a contextual conditioning and extinction paradigm provoking anxiety. Twenty-one healthy persons participated in a differential context conditioning procedure with two different background colours as contexts. During acquisition increased activity to the conditioned stimulus (CS+) relative to the CS− was found in the left hippocampus and anterior cingulate cortex (ACC). The amygdala, insula and inferior frontal cortex were differentially active during late acquisition. Extinction was accompanied by enhanced activation to CS+ vs. CS− in the dorsal anterior cingulate cortex (dACC). The results are in accordance with animal studies and provide evidence for the important role of the hippocampus in contextual learning in humans. Connectivity analyses revealed correlated activity between the left posterior hippocampus and dACC (BA32) during early acquisition and the dACC, left posterior hippocampus and right amygdala during extinction. These data are consistent with theoretical models that propose an inhibitory effect of the mPFC on the amygdala. The interaction of the mPFC with the hippocampus may reflect the context-specificity of extinction learning.

## Introduction

Interactions between neural structures involved in memory and emotion are fundamental in the adaptation to biologically and socially significant stimuli (LeDoux, 2000). Pavlovian fear conditioning is a form of associative learning, in which an originally neutral stimulus or context (e.g. a tone or a visual background) serves as conditioned stimulus (CS) and is presented together with an unconditioned threat stimulus (US). After several pairings the neutral cue evokes a phasic fear response and the context evokes a sustained anxiety response (Marks, 1987). Pavlovian fear conditioning has been widely used as a model of anxiety disorders (Bouton *et al.*, 2001; Myers & Davis, 2002), however, human neuroimaging studies have mainly focused on fear conditioning of discrete cues (Büchel *et al.*, 1998; LaBar *et al.*, 1998). Animal research indicates that the amygdala is involved in context and cue conditioning (LeDoux, 2000), whereas the dorsal hippocampus is central only for contextual memory formation (Kim & Fanselow, 1992; Anagnostaras *et al.*, 1999; Rudy *et al.*, 2002). In a positron emission tomography study, Hasler *et al.* (2007) used a cue and context to induce predictable fear or unpredictable anxiety. Amygdala activation was present only in the predictable and right hippocampal activation in the unpredictable condition, suggesting a unique involvement of the hippocampus in contextual anxiety.

Delayed extinction or the failure to extinguish acquired fear and anxiety responses may also be crucial for anxiety disorders (Milad *et al.*, 2006; Blechert *et al.*, 2007). Extinction involves the formation of new memories that inhibit, without actually erasing, the original conditioning trace (Myers & Davis, 2007). Human imaging studies support the role of the medial prefrontal cortex (mPFC), including the anterior cingulate cortex (ACC) and the amygdala in extinction learning of discrete CSs (Gottfried & Dolan, 2004; Phelps *et al.*, 2004). Likewise, imaging studies have implicated the amygdala, ventromedial PFC and hippocampus in extinction recall (Kalisch *et al.*, 2006; Milad *et al.*, 2007), providing evidence in line with animal research that this network contributes to contextual modulation of conditioned responses. Whether contextual extinction learning in humans follows similar neurobiological principles is unknown.

We used event-related functional magnetic resonance imaging (fMRI) to determine the role of the hippocampus, mPFC (including the ACC) and amygdala during acquisition and extinction learning of contextual anxiety in humans. We examined whether acquisition and extinction share common neural substrates, and analysed the functional coupling of involved brain structures. Context conditioning is thought to induce a state of sustained anxiety, as the appearance of the US is not clearly associated with the onset of a signalling cue and is thus enhanced by temporal unpredictability of the US (Grillon *et al.*, 2004; Vansteenwegen *et al.*, 2008). The present study used a contextual conditioning paradigm, in which the CS was a long-lasting background stimulus that illuminated the field of view of the participants with two different colours, and by delivering the US at differing time points during the presentation of one of the colours.

## Materials and methods

### Participants

Twenty-one right-handed (seven female) persons participated in the study (mean age 21.8 years, range 19–27 years). Exclusion criteria were nervous system or mental disorders and previous trauma exposure. Written informed consent was obtained from all participants prior to the study, which was approved by the Ethics Committee of the Medical Faculty Mannheim of the University of Heidelberg. The study conforms to the Code of Ethics of the World Medical Association (Declaration of Helsinki).

### Stimuli

#### Unconditioned threat stimulus

The US consisted of unpleasant, but tolerable electrical stimulation to the right thumb. An electrical stimulus generator (Digitimer, DS7A, Welwyn Garden City, UK) delivered the US through a cupric (copper) electrode. For each participant, the level of stimulation was individually determined. Participants were given a series of painful stimuli (duration 50 ms, 20 Hz), starting with a mild stimulus, which was gradually increased to the level the participants indicated as ‘painful’ (pain threshold). Then the stimulation was further increased to the level of pain tolerance. This procedure was repeated three times. Finally, we delivered stimuli that were 80% above pain threshold. Participants were asked to rate the intensity and unpleasantness of the pain on a Likert scale ranging from 0 = not painful or unpleasant to 10 = extremely painful or extremely unpleasant. For the experiment we used stimulation intensities that were rated at least 7 on average (intensity: M = 7.10, SD = 0.62, range = 6–8; unpleasantness: M = 7.33, SD = 1.32, range = 5–9).

#### Contextual stimulus

**Fig. 1:**
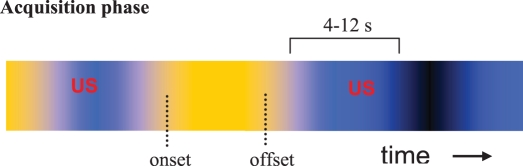
Schematic representation of the acquisition phase of the experiment. As can be seen, the colours are presented gradually until they reach their maximal spectrum. During habituation CS and US were presented in random order. During conditioning, the context changed gradually from orange to orange and blue to blue and orange. If the same colour was repeated (i.e. from blue to blue), the colour gradually changed from blue to black and from black to blue. In our example, the blue context represents the CS+ that was paired in the acquisition phase with a threat stimulus and later extinguished. Note that the onset of threat stimulus was unpredictable, i.e. it was given at varying intervals after context onset. During extinction no US was delivered. Within extinction, the colours were presented with clear on- and offsets, separated by a black screen.

In accordance with previous studies (Vansteenwegen *et al.*, 2005; Effting & Kind, 2007; Neumann *et al.*, 2007; Barrett & Armony, 2008), two different colours (orange and blue) were used to represent two different spatial contexts. As a modification, in the current study the individual colours were initially blended and then became increasingly clearer until they reached their full spectrum. After several seconds the colours were blended off and passed into the next colour. This gradual background colour was projected in the scanner via a mirror system and created the impression of a surround colour and thus of an actual context. The colours had a slow onset to reinforce the feeling of context, and the colour gradients were presented to produce a more complex processing of the stimuli. To produce colour gradients we used a fixed-speed transition from one point to the next on a straight line in the red-green-blue colour space in a linear fashion. The colours reached their maximal spectrum between 3 and 4 s, and continued from 1 to 8 s. The duration of the CSs (including the gradients) varied randomly between 4 and 12 s. In 50% of the trials, one of the contexts was paired with shock (CS+), the other context, designated as CS−, was never paired with shock. A 50% partial reinforcement schedule was used to investigate the haemodynamic activities to the CS uncontaminated by the US (cf. Büchel *et al.*, 1998; Büchel *et al.*, 1999). The US was delivered at unpredictable time points within the time frame of the CS± after the maximal spectrum of the colour was achieved to maximize unpredictability (Grillon *et al.*, 2006). The stimuli were presented in pseudorandomized order, and the assignment of colours as CS+ was counterbalanced across participants. During habituation a black screen was shown for 4–12 s (random variation) between the colour presentations, and the CSs and the US were presented in random order. During acquisition the colours on the screen changed gradually between orange and blue and blue and orange in random order (Fig. 1). In the extinction phase there was a clear on- and offset of the two colours and the separation of them by a black screen in order to obtain a better onset for the CS-related blood oxygen level-dependent (BOLD) effects, which were expected to be weaker during extinction as the US was omitted.

## Experimental procedure

Throughout the experiment participants looked through a mirror mounted on the head coil where they viewed the coloured screen, presented by a projector. Participants were not informed about the CS–US contingency and were told to passively view the stimuli.

During habituation the CSs and the US were presented 10 times in random order. The duration of the US was 2.9 s, and the interstimulus interval ranged from 4 to 12 s (mean 8 s). The two acquisition phases consisted of five CS+ without US (CS+_unpaired_), five CS+ with US (CS+_paired_), and 10 CS− (safe condition). The delay of CS+ and US varied between 3 and 8 s after stimulus onset, i.e. the US was unpredictable for participants. In the following extinction block, conditioned anxiety was extinguished by presenting 10 CS+ without US and 10 CS−. After each block, participants rated the valence and arousal of the CSs and the CS–US contingency on scales ranging from 1 = very calm to 9 = very arousing, 1 = very pleasant to 9 = very unpleasant, and 1 = no CS–US contingency to 9 = perfect CS–US contingency. The subjective ratings were obtained by presenting the relevant background colour and presenting standardized rating instructions via headphones. Participants made their ratings by speaking into a microphone attached to the headphones. All other instructions for the experimental procedure were also presented via headphones.

## Data analysis

### Functional imaging

Data were acquired with a 1.5 Tesla Magnetom VISION whole body MR-scanner (Siemens Medical Solutions, Erlangen, Germany) equipped with a head volume coil. Contiguous transversal T2* weighted echoplanar images (EPIs) with BOLD contrast were used (echo time 45 ms, flip angle 90°) that covered the whole brain (35 slices, slice thickness 3 mm, 1 mm gap, FOV 220 × 220 mm, in-plane resolution 3.44 × 3.44 mm). The effective repetition time (TR) was 3.77 s/volume. A total of 390 volumes (habituation: 130; acquisition I and II: 80 each; extinction: 100) were recorded. The first three images were discarded to allow steady state magnetization. Statistical parametric mapping software was used for image processing and analysis (Statistical Parametric Mapping Software, SPM2, Wellcome Department of Imaging Neuroscience, London, UK). The images were slice-time and motion-corrected, spatially normalized to the Montreal Neurological Institute (MNI) EPI template, spatially smoothed with a Gaussian kernel of 10 mm at full-width half-maximum, temporally high-passed filtered (cutoff 128 s), and corrected for autocorrelations using first-order autoregressive modelling. Specific effects were tested by applying linear contrasts to the parameter estimates for each event. Contrast images of interest were calculated for each subject (CS+_unpaired_, CS−), and the resulting contrast images were entered into a second-level random model (random-effects) in SPM2 via a repeated-measures analysis of variance (anova) with non-sphericity correction to produce group results.

For each participant, condition-specific regressors were defined (CS+_unpaired_, CS−). The regressors were modelled as two separate box car regressors. The duration of each regressor was 4–8 s. The onset was defined as the time point when colours reached their maximal spectrum. The duration of each regressor (which was identical for the CS+_unpaired_ and CS−) was defined from the onset of the maximal spectrum to the time point until the colour was blended off. Each regressor was convolved with a canonical haemodynamic response function (HRF). These regressors were used in a general linear model (multiple regression) of brain activation at each voxel to yield parameter estimates of the contribution of each regressor to the fMRI signal measured in each voxel. Voxelwise contrast effects were then calculated to produce within-subject estimates of effects of interests using the contrast CS+_unpaired_ vs. CS− for early and late acquisition, and extinction. To assess activation specific to acquisition and extinction learning, we used the contrasts acquisition vs. extinction and extinction vs. acquisition. Furthermore, common brain areas involved in both acquisition and extinction learning were analysed by using a conjunction analysis.

According to our *a priori* hypothesis we used a region of interest (ROI) approach for the amygdala, hippocampus and ACC. These regions were specified using an established anatomical atlas (Tzourio-Mazoyer *et al.*, 2002) to define and create anatomically based ROIs using the masks for regions of interest analysis (MARINA) software program (Bertram Walter Bender Institute of Neuroimaging, University of Giessen, Germany). The ROIs were applied using the ‘small volume correction’ option in SPM2 with a threshold of *P*<0.05. The false discovery rate (FDR) was applied to correct the data for multiple comparisons (Benjamini & Hochberg, 1995), and the extent threshold was five contiguously active voxels (2 × 2 × 2 mm^3^). For the entire brain we used a threshold of *P*<0.05 (FDR-corrected) with an extent threshold of *k*=10 voxels.

For assessment of functional coupling, we used a correlation analysis (e.g. Heinz *et al.*, 2004). Based on animal studies functional coupling between the seed regions amygdala and hippocampus for early and late acquisition and mPFC for extinction were defined. Based on the actual activation patterns of our analysis (see Table 1) the following coordinates were employed as seed regions: left posterior hippocampus (MNI coordinates [−33, −36, −6]) for the early acquisition phase, left amygdala (MNI coordinates [−30, −3, −12]) and left dorsal hippocampus (MNI coordinates [−36, −21, −9]) for the late acquisition phase, and dACC (BA32; MNI coordinates [12, 36, 24]) for the extinction phase. fMRI time series were extracted and used as regressors in a subsequent single-subject analysis. These contrasts were used to carry out a random effects analysis to determine functional coupling using one-sample *t*-tests.

**Table 1:** Brodmann areas and MNI coordinates of activations to CS+ vs. CS− during the early and late acquisition and extinction phase

				MNI coordinate			
Brain region (and hemisphere)	BA	Cluster size (number of voxels)	*Z*-value	*x*	*y*	*z*	*P*-value
Early acquisition							
Posterior hippocampus (L)		23	3.48	−33	−36	−6	< 0.05*
Late acquisition							
Inferior frontal gyrus (R)	BA9/47	737	5.56	54	18	0	< 0.05
Insula (R)	BA13	110	4.71	−39	21	0	< 0.05
Medial superior frontal gyrus (R)	BA8	67	4.42	3	33	51	< 0.05
Supramarginal gyrus (R)	BA40	332	4.77	66	−36	39	< 0.05
Ventral putamen (L) extending to amygdala		218	4.58	−30	−3	−9	< 0.05
Amygdala (L)		24	4.22	−30	−3	−12	< 0.05*
Dorsal hippocampus (L)		12	3.11	−36	−21	−9	< 0.05*
Primary somatosensory cortex (L)	BA40	132	3.99	−57	−30	48	< 0.05
Extinction							
Superior frontal gyrus (R)	BA10	47	3.15	36	54	15	< 0.05
Superior frontal gyrus (L)	BA10	34	3.08	−18	48	15	< 0.05
Dorsal anterior cingulate (R)	BA32	15	2.98	12	36	24	< 0.05*

L, left hemisphere; R, right hemisphere; BA, Brodmann area; MNI, Montreal Neurological Institute.

* FDR-corrected for the ROI.

### Skin conductance response (SCR) and self-report data

SCR was recorded by VarioPort (BECKER MEDITEC, Karlsruhe, Germany) at a sampling rate of 16 Hz from electrodes placed on the thenar and hypothenar eminence of the participants’ right hand. Values for SCR amplitudes were converted to be normally distributed [ln (1 + SCR)]. SCR amplitudes were determined as maximum reaction occurring 1–4 s after maximal colour onset was reached, and were measured in microsiemens (mS). Analyses were performed using EDA-PARA software (F. Schäfer, Wuppertal, Germany). The procedures followed the guidelines by Fowles *et al.* (1981). Extreme cases were detected by simple boxplot summaries and were individually replaced by the mean of the adjacent values. Due to technical artefacts SCR data of four persons in the late acquisition phase and of six persons in the habituation, early acquisition and extinction phases each had to be excluded from the analyses. SCR and self-report data were analysed per phase in accordance with the imaging data using *t*-tests for dependent measures with hypothesis-based one-tailed levels of significance (*P* < 0.05).

## Results

### SCR

**Fig. 2:**
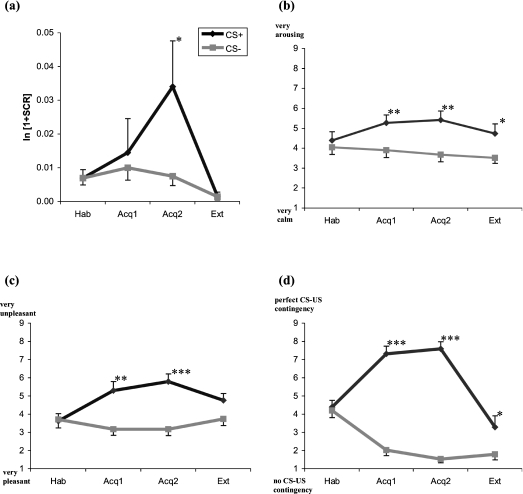
Skin conductance response (SCR; means and standard errors of the means) to the CS+_unpaired_ vs. CS− for the four phases (habituation, first and second acquisition phase, and extinction) during differential context conditioning (a). (b–d) Self-report data (means and standard errors of the means) for arousal (b), valence (c), and CS–US contingency (d). *P < 0.05, **P < 0.01, ***P < 0.001.

In the late acquisition phase SCRs to the CS+_unpaired_ were significantly higher than to the CS− (*t*_16_ = 1.93, *P* < 0.05). No significant CS+/CS− differentiation was found for the habituation, early acquisition and extinction phase (Fig. 2a).

### Self-report data

Arousal ratings were significantly higher for the CS+ than CS− after early (*t*_20_ = 2.81, *P* < 0.01) and late acquisition (*t*_20_ = 3.37, *P* < 0.01) and extinction (*t*_20_ = 2.54, *P* < 0.05; Fig. 2b), but not for habituation. Valence ratings to the CS+ vs. CS− differed significantly for both acquisition phases (early: *t*_20_ = 3.13, *P* < 0.01; late: *t*_20_ = 4.34, *P* < 0.001; Fig. 2c), but not during extinction (*t*_20_ = 1.22, *P* = 0.32) or habituation (*t*_20_ = 1.42, *P* = 0.29), indicating increased reports of unpleasantness to the CS+ relative to the CS− during learning. Contingency ratings were significantly higher for the CS+ vs. CS− after early (*t*_20_ = 8.43, *P* < 0.001) and late acquisition (*t*_20_ = 12.00, *P* < 0.001), and during extinction (*t*_20_ = 2.74, *P* < 0.05; Fig. 2d), but not after the habituation phase (*t*_20_ = 1.03, *P* = 0.42).

### fMRI

#### Brain activation during acquisition

**Fig. 3:**
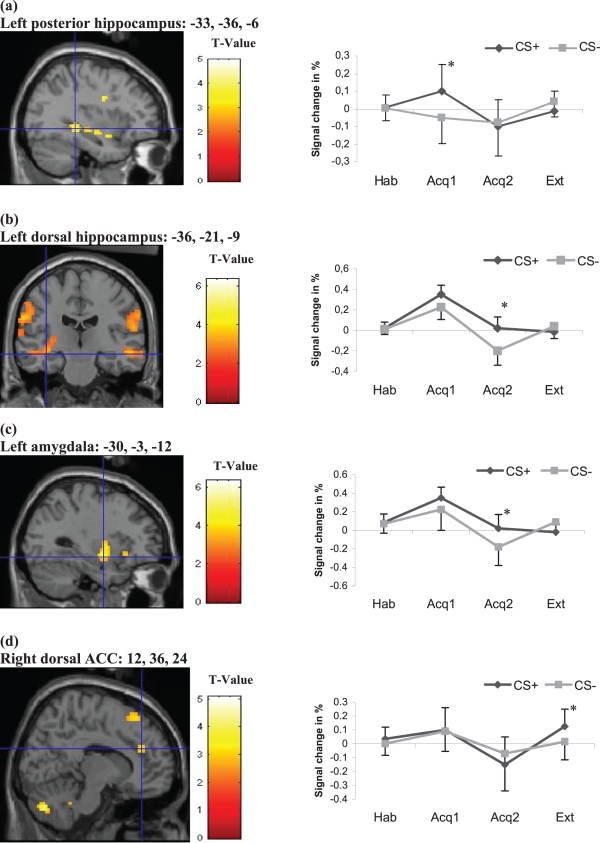
Neural activation and percent signal changes for the single conditioning phases (Hab = habituation; Acq1 = early acquisition phase; Acq2 = late acquisition phase; Ext = extinction) to CS+ vs. CS− for (a) the left posterior hippocampus, (b) the left dorsal hippocampus (during late acquisition), (c) the left amygdala and (d) the right dorsal anterior cingulate cortex (dACC; BA32). *Significant CS+/CS− differentiation.

During habituation the BOLD signal did not significantly differ between CS+_unpaired_ and CS−. Throughout the two acquisition phases, significant BOLD responses were observed in a distributed prefrontal network (mPFC, orbitofrontal cortex and supplementary motor cortex), as well as the left amygdala and discrete hippocampal areas (see Supplementary Table S1). Specifically, during the early acquisition phase the BOLD signal was significantly more pronounced to CS+_unpaired_ relative to CS− in the left posterior hippocampus (Fig. 3a), whereas during late acquisition the left dorsal hippocampus and the left amygdala (Fig. 3b and c), as well as the left ventral putamen, the right insula, the right supramarginal gyrus and right inferior prefrontal cortex (Table 1) responded significantly stronger to CS+_unpaired_ than to CS−.

#### Brain activation during extinction learning

In contrast to acquisition, no increased hippocampal activity to CS+ relative to CS− was observed during extinction. Instead, significantly increased activation was found in the prefrontal cortex, i.e. the left and right superior frontal gyri and the right dorsal anterior cingulate cortex (dACC; BA32; Table 1 and Fig. 3d). This brain area was specific to extinction learning, as the contrast between extinction and late acquisition revealed significant BOLD responses in the dACC (BA32) only during extinction. In addition, significant BOLD responses in the left medial frontal gyrus (BA9) and the left precuneus (BA19) were associated with extinction only (Table 2). Separate analysis of early and late extinction revealed no significant differences in brain activity. In both extinction phases significant activity was found in the dACC (BA32).

**Table 2:** Differential brain activations to CS+ vs. CS− for extinction vs. acquisition phases and vice versa

				MNI coordinate			
Brain region (and hemisphere)	BA	Cluster size (number of voxels)	*Z*-value	*x*	*y*	*z*	*P*-value
Early acquisition > late acquisition							
Superior frontal gyrus (L)	BA8	30	3.04	−18	39	45	< 0.05
Parahippoccampal gyrus (L)		18	3.31	−24	−30	−21	< 0.05*
Late acquisition > early acquisition							
Inferior frontal gyrus (R)	BA47	7	3.68	27	27	−6	< 0.05
Inferior frontal gyrus (R)	BA45	13	3.44	54	18	6	< 0.05
Inferior frontal gyrus (L)	BA47	23	3.34	−36	24	−3	< 0.05
Late acquisition > extinction							
Amygdala (L)		119	3.23	−27	−6	−12	< 0.05*
Extinction > late acquisition							
Dorsal anterior cingulate (R)	BA32	7	2.83	12	36	21	< 0.05*
Medial frontal gyrus (L)	BA9	93	3.05	−6	54	15	< 0.05*
Precuneus (L)	BA19	34	3.63	−39	−75	42	< 0.05

L, left hemisphere; R, right hemisphere; BA, Brodmann area; MNI, Montreal Neurological Institute.

* FDR-corrected for the ROI.

### Connectivity analysis

#### Functional coupling during the acquisition phase

During early acquisition we found significant functional coupling between the left posterior hippocampus (seed region) and the bilateral orbitofrontal cortex, the right rostral anterior cingulate, the bilateral precuneus and the contralateral hippocampus. During late acquisition increased activation of the left amygdala (seed region) correlated significantly with enhanced BOLD responses in the bilateral inferior and superior orbitofrontal cortex, bilaterally in the insula and the hippocampus. For the dorsal hippocampus as seed region significant correlations with the contralateral hippocampus (dorsal and posterior), amygdala, middle orbitofrontal cortex, inferior orbitofrontal cortex, right putamen and right insula were found.

#### Functional coupling during extinction

**Fig. 4:**
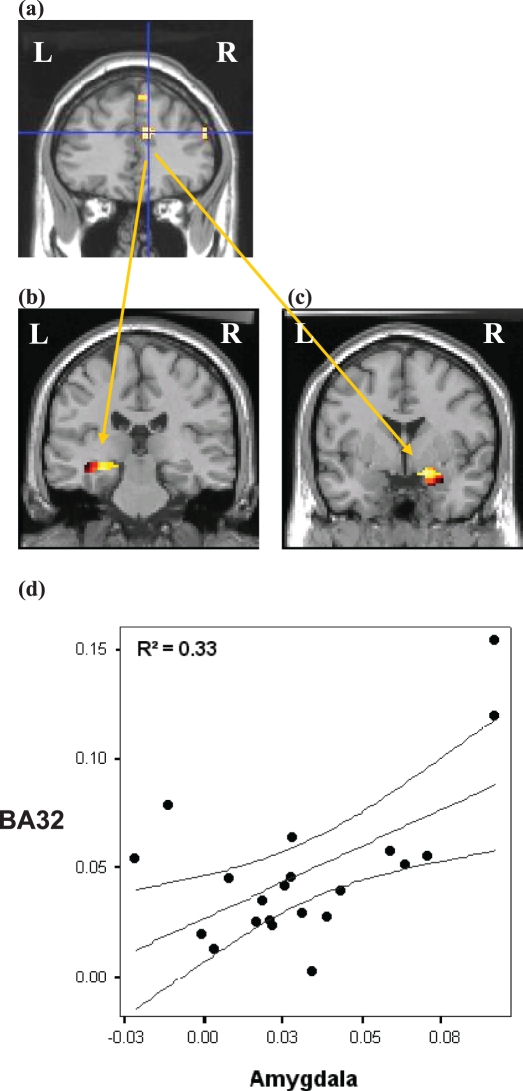
T-maps for extinction showing significant coupling between (a) BA32 (seed region) and (b) left posterior hippocampus [MNI coordinate (−27, −24, −9) and (−24, −18, −12), *P* < 0.05, FDR-corrected for ROI] and (c) right amygdala [MNI coordinate (18, 0, −15), *P* < 0.05, FDR-corrected for ROI]. (d) Regression line between signal changes to CS+ vs. CS− in BA32 (seed region) and right amygdala (target region). BA, Brodmann area; L, left; R, right.

During the extinction phase, increased activity in BA32 (seed region) covaried significantly with BOLD responses in the left posterior hippocampus, right amygdala (Fig. 4) and the prefrontal and orbitofrontal cortices. Detailed information on the coordinates of the significant brain regions resulting from connectivity analyses are displayed in Table 3.

**Table 3:** Areas showing positive functional coupling with the specified seed regions

			MNI coordinate			
Brain region (and hemisphere)	BA	*Z*-value	*x*	*y*	*z*	*P*-value (FDR- corrected)
Early acquisition (seed region left posterior hippocampus)						
Middle orbitofrontal cortex (L)	BA10	4.48	−33	48	−6	< 0.05
Inferior orbitofrontal cortex (L)	BA10	4.24	−36	39	−12	< 0.05
Medial orbitofrontal cortex (R)	BA10	4.26	9	51	−9	< 0.05
Anterior cingulate cortex (R)	BA32	3.57	15	36	6	< 0.05
Precuneus (R)	BA23	3.92	9	−51	21	< 0.05
Precuneus (L)	BA23	3.41	0	−69	27	< 0.05
Globus pallidum (R)		4.40	21	−3	3	< 0.05
Dorsal hippocampus (R)		3.22	33	−27	−9	< 0.05*
Superior temporal gyrus (R)	BA21	3.61	54	3	−15	< 0.05
Inferior temporal gyrus (R)	BA37	3.57	42	−42	−12	< 0.05
Late acquisition phase (seed region left amygdala)						
Inferior orbitofrontal gyrus (L)	BA47	4.39	−30	33	−18	< 0.05
Inferior orbitofrontal gyrus (R)	BA47	4.06	30	33	−15	< 0.05
Superior frontal gyrus (L)	BA6	4.07	−24	−3	63	< 0.05
Supplementary motor cortex (L)	BA6	3.45	3	31	63	< 0.05
Putamen (L)		5.02	−33	−15	−9	< 0.05
Hippocampus (L)		4.93	−27	−33	−9	< 0.05*
Hippocampus (R)		4.66	24	−33	−6	< 0.05*
Insula (L)	BA13	4.62	−24	12	−18	< 0.05
Insula (R)	BA13	5.32	39	−6	−6	< 0.05
Inferior temporal gyrus (R)	BA37	3.56	45	−48	−15	< 0.05
Precuneus (L)	BA19	4.81	−33	−81	39	< 0.05
Extinction phase (seed region BA32)						
Middle frontal gyrus (L)	BA32	5.40	−18	39	24	< 0.05
Middle frontal gyrus (R)	BA10	5.24	24	48	24	< 0.05
Medial orbitofrontal gyrus (R)	BA10	3.82	0	45	−9	< 0.05
Anterior cingulate (R)	BA24	5.40	0	27	18	< 0.05
Amygdala (R)		2.91	18	0	−15	< 0.05*
Posterior hippocampus (L)		3.14	−27	−24	−12	< 0.05
Posterior hippocampus (L)		3.03	−24	−18	−12	< 0.05
Middle temporal gyrus (R)	BA39	4.05	51	−72	18	< 0.05

L, left hemisphere; R, right hemisphere; BA, Brodmann area; MNI, Montreal Neurological Institute.

* FDR-corrected for the ROI.

## Discussion

The present study examined the neural correlates of early and late acquisition, as well as extinction of fear in a contextual conditioning paradigm. In addition, connectivity analyses were employed to identify interactions of the mPFC, amygdala and the hippocampus during contextual acquisition and extinction learning. During preparation of the present manuscript two fMRI studies were published that also have assessed context onditioning (Alvarez *et al.*, 2008; Marschner *et al.*, 2008). Marschner *et al.* (2008) used a combined cue and context conditioning paradigm with background pictures as contexts. Alvarez *et al.* (2008) used a virtual reality precedure to create contexts. In line with the present study, both studies delivered unpredictable threat stimuli, which were associated to one context.

In the current study, self report data showed a differential conditioning effect in both acquisition phases. Significant activation of the amygdala to CS+ vs. CS− and differential SCRs occured only in the late acquisition phase. Our results are in contrast to cue conditioning where much faster differential SCR and amygdala activation have been observed that also showed fast habituation (Büchel *et al.*, 1998; Birbaumer *et al.*, 2005; Wessa & Flor, 2007; Marschner *et al.*, 2008). Alvarez *et al.* (2008) reported fast attenuation of amygdala responses, and Marschner *et al.* (2008) found no amygdala activation in their context conditioning paradigm. In both studies very long contexts (28 and 60 s, respectively) were used.

In line with animal studies (Phillips & LeDoux, 1992; Maren *et al.*, 1997; Fanselow, 2000), significant hippocampal activation emerged during contextual conditioning in our study. The left posterior hippocampus was activated more intensely during early conditioning, whereas in late acquisition significant BOLD responses were found in the left dorsal hippocampus. Our data are in accordance with Alvarez *et al.* (2008) who also reported hippocampal activity during acquisition, although they found activity in the right anterior hippocampus. In line with the present study, Marschner *et al.* (2008) reported activity in the left hippocampus, which was specific for context conditioning. However, both studies observed a rapid attenuation of hippocampal activity over time, which is in contrast to our results. These differences may be as a result of the longer-lasting contexts used in their studies, or the fact that no separate analyses for early and late acquisition were performed. Indeed, we found that the posterior hippocampus was specific for the early acquisition, whereas the dorsal hippocampus was significantly activated only in late acquisition. However, conjunction analyses revealed dorsal hippocampal activity during both acquisition phases, indicating that the dorsal hippocampus is also active during early acquisition, albeit non-significant. Our findings are also in line with the study of Hasler *et al.* (2007), who presented aversive stimuli related to cues (predictable) and contexts (unpredictable). The authors also observed selective dorsal hippocampal activation during the context that signalled unpredictable shock, although they did not use a context conditioning design (see also Ploghaus *et al.*, 2001). In contrast, two imaging studies using a combined cue and context paradigm failed to find hippocampal activity (Armony & Dolan, 2001; Milad *et al.*, 2007). However, the authors used predictable threat stimuli instead of unpredictable USs. It is known that context conditioning is enhanced by temporal unpredictability of the US (Grillon *et al.*, 2004; Vansteenwegen *et al.*, 2008). Indeed, several studies have shown that with unpaired CS–US presentations the context becomes the only predictor, leading to increased context conditioning (Rescorla & Wagner, 1972; Grillon & Davis, 1997; Grillon, 2002).

In sum, the present data provide clear evidence of the role of the hippocampus in human context conditioning. Moreover, the findings of the current study extend previous results showing different hippocampal activations during early and late context conditioning. However, it still needs to be determined to what extent dorsal and posterior areas of the hippocampus are involved in different functions. It is possible that early posterior activation, equivalent to the dorsal hippocampal activation in rats, is related to the analysis and association of the contextual spatial stimuli whereas the later dorsal activation might involve memory consolidation (Kim & Fanselow, 1992; Manns *et al.*, 2003).

Context conditioning also involved the inferior frontal cortex, the insula and the parietal cortex, as well as the ventral putamen. Rodent studies have shown that lesions of these regions (except the parietal cortex) result in impairment of contextual fear (Morgan & LeDoux, 1999). Activation in the insula was also reported in the study of Marschner *et al.* (2008). Thus, these results implicate a network of brain regions that contribute to contextual conditioning.

Concerning connectivity between brain structures, the results demonstrated significant functional coupling between the posterior hippocampus and prefrontal cortex, notably the mPFC (BA32) and medial frontal gyrus (BA10) during early acquisition. These findings are in accordance with the assumption that the hippocampus is important for the encoding of contextual memories that may be stored in prefrontal areas (Lavenex & Amaral, 2000). During late acquisition there was a positive correlation between left amygdala activation and bilateral activation in the orbitofrontal (BA11) and inferior frontal gyrus (BA47) as well as the hippocampus and the insula. The activation of the left dorsal hippocampus correlated significantly with the activation of amygdala, middle and inferior orbitofrontal gyrus, right putamen and right insula. The involvement of frontal cortical regions in the modulation of amygdala reactivity and the mediation of effective emotion regulation has been shown in several studies (e.g. Birbaumer *et al.*, 2005; Stein *et al.*, 2007). The results are also in line with Alvarez *et al.* (2008), who indicated that the medial amygdala was the source of key efferent and afferent connections including input from the orbitofrontal cortex. The insula has close connections to the amygdala and is involved in the processing of the arousal value of emotional sitmuli (Craig, 2003; Anders *et al.*, 2004; Pollatos *et al.*, 2007). The functional coupling of amygdala and hippocampus has previously been described in both animal (Rudy *et al.*, 2004) and human studies (Smith *et al.*, 2005). Smith *et al.* (2004) also found an enhanced connectivity between the hippocampus and amygdala during the retrieval of emotionally valenced contextual information. It is supposed that the amygdala responds to and processes emotional information retrieved from hippocampus-dependent, i.e. contextual, memory. Furthermore, hippocampus–amygdala effects were observed in the left hemisphere, consistent with previous studies of autobiographical memory (Maguire, 2001; Greenberg *et al.*, 2005), as well as explicit processing (Glaescher & Adolphs, 2003) or retrieval (Smith *et al.*, 2005) of emotional information.

In addition to the examination of the acquisition of context conditioning, a second goal of the present study was to investigate contextual extinction learning. Extinction can be separated in two processes: extinction learning and consolidation (extinction memory). A prevalent theoretical model presumes that inhibitory influences of the mPFC on the amygdala might suppress the expression of conditioned fear (Sotres-Bayon *et al.*, 2004). To date, there are no imaging studies, which have assessed contextual fear extinction in humans *per se*. Previous imaging studies related to extinction learning have focused only on discrete CSs (Gottfried & Dolan, 2004; Phelps *et al.*, 2004). During extinction learning we observed mPFC (BA32) activation to the CS+ relative to the CS−, which was specific for the extinction phase. Our data are in line with animal data and human neuroimaging studies providing evidence that the mPFC regulates the expression of fear and anxiety by inhibiting the amygdala (Milad & Quirk, 2002; Maren & Quirk, 2004; Sotres-Bayon *et al.*, 2004; Milad *et al.*, 2006). Recent fMRI studies using simple cues as CS reported activity in the mPFC also in extinction learning (Gottfried & Dolan, 2004; Phelps *et al.*, 2004; Milad *et al.*, 2007). In contrast to many studies, we did not observe significant amygdala activation during extinction learning. This is surprising because the amygdala is considered to be important for active extinction learning (Quirk *et al.*, 1995; LaBar *et al.*, 1998; Hobin *et al.*, 2003; Phelps *et al.*, 2004; Milad *et al.*, 2007). On the other hand, even the animal literature did not find consistent evidence for amygdala activation during early extinction (see Myers & Davis, 2007; for review). We also failed to find hippocampal activation during extinction learning. Animal studies confirm the important role of the dorsal hippocampus in both the acquisition and initial extinction of conditioned fear (Phillips & LeDoux, 1992; Corcoran *et al.*, 2005; Pentkowski *et al.*, 2006; Ji & Maren, 2007). However, our connectivity analysis suggested that extinction learning was mediated by a network of brain areas, including the mPFC, hippocampus and right amygdala. This interaction of the mPFC with the hippocampus during extinction learning may indicate the establishment of a context-specific extinction memory in prefrontal cortex (Corcoran & Quirk, 2007). Additionally, the functional coupling of the mPFC with the amygdala might be indicative of the inhibition of the amygdala in the actual context. Our data are consistent with previous animal studies showing an interaction of the amygdala and the mPFC during extinction (cf. Sotres-Bayon *et al.*, 2004, 2006). Kalisch *et al.* (2006) and Milad *et al.* (2007) found a similar network in their studies. However, they examined recall of extinction memory on a later day rather than initial extinction learning. The authors concluded that this network specifically reflects context-dependent recall of extinction memory, as such coupling was only found in the extinction phase but could not be observed in the early and late acquisition phases.

The study has several limitations. First, our study had a 3 : 1 ratio of males to females. The higher prevalance of females than males may have led to different results than a gender-matched sample would have provided. However, we found no significant gender differences in conditioning of the verbal measures. Potential gender effects in context conditioning need to be examined in future studies. Second, the use of virtual reality contexts such as used by Alvarez *et al.* (2008) might be helpful in optimizing fMRI protocols, which would permit a comparative analysis of cue and context in one paradigm (cf. Baas *et al.*, 2004). Third, the connectivity analysis merely permits an evaluation of correlations between regions, but not an analysis of the direction of the response as in structural equation modelling or a differential analysis of inhibitory or excitatory connections as in independent component analyses. These questions need to be addressed in future research.

Despite these limitations, we believe that this study provides important insights into the neural mechanisms and interactions of context-dependent conditioning and contextual extinction learning. To our knowledge this is the first study that examined the neural correlates and functional coupling of brain areas of contextual extinction learning. Our data provide evidence that in humans the hippocampus and amygdala are important in contextual acquisition, and that these brain structures are functionally coupled with prefrontal regions. During early extinction learning, which immediately follows the acquisition phase, a network consisting of the mPFC, hippocampus and amygdala seems to regulate the extinction of sustained anxiety.

Understanding the neural mechanisms of contextual conditioning and extinction has important implications for the treatment of humans with anxiety disorders, such as posttraumatic stress disorder. Cognitive-behavioural therapy in humans is based on extinction and typically involves exposure to fear-eliciting cues in safe settings (Foa *et al.*, 1999). However, contextual restrictions on extinction can considerably complicate anxiety therapy, resulting in recall of fear memory in a new contextual setting although extinction was successful in the therapeutic context. In addition, the hippocampal and prefrontal deficits observed in persons with anxiety disorders such as posttraumatic stress disorder suggest that the acquisition of contextual information might be impaired and that this might contribute to deficient cue extinction as previously shown (Orr *et al.*, 2000; Wessa & Flor, 2007). Thus, a better understanding of contextual conditioning may yield important insights for both the development and the treatment of anxiety disorders.

## Abbreviations

ACC: anterior cingulate cortex
BOLD: blood oxygen level dependency
CS: conditioned stimulus
dACC: dorsal anterior cingulate cortex
EPI: echoplanar images
FDR: false discovery rate
fMRI: functional magnetic resonance imaging
HRF: haemodynamic response function
MNI: Montreal Neurological Institute
mPFC: medial prefrontal cortex
ROI: region of interest
SCR: skin conductance responses
US: unconditioned stimulus

## Supporting Information

Table S1. Conjunction analysis for CS+ vs. CS− during early and late acquisition.
