# Supplementary material for: Context conditioning and extinction in humans: differential contribution of the hippocampus, amygdala and prefrontal cortex
Source: Eur J Neurosci. 2009 Feb;29(4):823–32. doi: 10.1111/j.1460-9568.2009.06624.x (PMC2695154; doi:10.1111/j.1460-9568.2009.06624.x)
Supplement: Supplementary file 1 [file ejn0029-0823-SD1.doc]

**Table S1. Conjunction analysis for CS+ versus CS- during** early and late acquisition

|  |  | Cluster size |  | MNI coordinate | | |  | |
| --- | --- | --- | --- | --- | --- | --- | --- | --- |
| Brain Region | BA | (voxels) | *Z*-value | *x* | *y* | *z* | | *P*-value |
| **Early and late acquisition** | | | | | | | | |
| Medial superior frontal gyrus (R) | BA8 | 136 | 5.44 | 3 | 33 | 51 | | < 0.05 |
| Inferior frontal gyrus (R) | BA45 | 1315 | 6.58 | 54 | 18 | 3 | | < 0.05 |
| Inferior/orbitofrontal gyrus (R) | BA47 | 593 | 5.47 | 48 | 39 | -12 | | < 0.05 |
| Inferior/orbitofrontal gyrus (L) | BA47 | 634 | 5.44 | -18 | 9 | -15 | | < 0.05 |
| Supplemental motor area (R) | BA8 | 201 | 4.59 | 6 | 21 | 45 | | < 0.05 |
| Supramarginal gyrus (R) | BA40 | 136 | 5.94 | 66 | -42 | 30 | | < 0.05 |
| Putamen (L) extending to the amygdala |  | 634 | 5.89 | -30 | -6 | -9 | | < 0.05 |
| Amygdala (L) |  | 36 | 5.60 | -30 | -3 | -12 | | < 0.05* |
| Dorsal hippocampus (L) |  | 33 | 4.09 | -33 | -12 | -12 | | < 0.05* |
| Dorsal hippocampus (L) |  | 33 | 3.83 | -36 | -18 | -12 | | < 0.05* |
| Hippocampus (L) |  | 33 | 3.08 | -36 | -27 | -9 | | < 0.05* |

L, left hemisphere; R, right hemisphere; BA, Brodmann area; MNI, Montreal Neurological Institute. *FDR-corrected for the ROI.
